# Students’ Inattention Symptoms and Psychological Need Satisfaction During the Secondary School Transition: The Protective Role of Teachers’ Involvement

**DOI:** 10.1177/10870547221105061

**Published:** 2022-06-14

**Authors:** Stéphane Duchesne, André Plamondon, Catherine F. Ratelle

**Affiliations:** 1 Laval University, Québec, QC, Canada

**Keywords:** attention problems, psychological needs, anxiety, aggression, school transition

## Abstract

**Objective:**

This study examined the prospective relationship from student inattention symptoms to changes in their psychological need satisfaction (PNS) during their transition to secondary school. In doing so, it has explored whether this temporal association was moderated by teachers’ involvement (TI).

**Method:**

A sample of 688 students (46% male; *M*_age_ = 11.82) followed in Grade 6 and Secondary 1 was selected from a stratified random list.

**Results:**

Inattention symptoms predicted a decrease in autonomy and competence need satisfaction, after adjusting for gender, anxiety, aggression, and PNS at baseline. In addition, TI in Secondary 1 attenuated the association between inattention and autonomy need satisfaction decline. TI also predicted a smaller decrease in competence need satisfaction, over and above the contribution of inattention.

**Conclusions:**

Results support the importance of TI in PNS of students who are struggling with inattention throughout a critical transition. Implications for educational practices and research are discussed.

Self-determination theory (SDT; Ryan & Deci, 2017) sustains that basic psychological need satisfaction (PNS) is essential for students’ academic motivation and learning. Research has identified social and contextual factors that promote students’ PNS such as teachers’ involvement (Froiland et al., 2019; Zhang et al., 2011). However, empirical knowledge is sparse about students’ personal vulnerability factors that may hamper their capacity to fulfill their needs (see Ryan & Deci, 2017). In this regard, it seems legitimate to examine the role of students’ inattention traits or symptoms, which are common in adolescence (Vergunst et al., 2019) and predict educational impairment (Fried et al., 2016). Based on SDT, the present prospective study investigated the contribution of students’ inattention symptoms at the end of elementary school as predictors of changes in PNS during the primary-secondary school transition, while exploring perceived teachers’ involvement in the classroom as a potential moderator.

## PNS and Secondary School Functioning

SDT, an empirically supported motivational theory, describes why individuals engage and persist in goal-oriented activities and how the environment can support them in doing so. One of the core propositions of this theory is that individuals’ functioning is optimal when their psychological needs for autonomy (experiencing volition and feeling that thoughts and actions are self-endorsed), competence (experiencing mastery and feeling efficacious in activities), and relatedness (experiencing connection, nurturing, and reciprocity with significant others) are fulfilled (Vansteenkiste et al., 2020). These three needs are assumed to operate in an interdependent way such that insatisfaction or frustration of any one of them can substantially thwart individuals’ optimal functioning in their daily activities (Ryan & Deci, 2017).

A great deal of research in education has shown that students’ PNS was associated with several positive indicators of functioning in secondary school settings such as intrinsic motivation (Gnambs & Hanfsting, 2016), concentration during lessons (Erturan-İlker et al., 2018), engagement in learning activities (Jang et al., 2010), academic (doing the required work) and social (making friends) adaptation (Duchesne et al., 2017), academic achievement (Earl et al., 2019), persistence intentions (Vallerand et al., 1997), well-being (Rodríguez-Meirinhos et al., 2020), and quality of life (Gillison et al., 2008). However, results show heterogenous trajectories of students’ perceived PNS at the end of elementary school and throughout the secondary school years (Ratelle & Duchesne, 2014). These trajectories were particularly detrimental for students whose PNS was consistently low or declining: the latter report lower levels of academic, social, and emotional adjustment at the end of high school, compared to students with higher or increasing levels of PNS. A better understanding of factors associated with the stability and change in PNS might therefore be useful to support at-risk students.

At least two main observations can be made from this body of literature. First, students’ PNS appears supports optimal functioning in secondary school. Second, many students feel that their psychological needs are not fully met when they are transitioning to secondary school. These observations have important implications for students with specific vulnerabilities that may compromise the fulfillment of their basic psychological needs and, subsequently, the quality of their school experience (Ryan & Deci, 2017). This particularly applied to students struggling with attention problems.

## Inattention Symptoms and PNS at School

Symptoms of inattention are part of the clinical presentation of ADHD, a neurodevelopmental condition that affects 5% to 7% of school-aged children and adolescents (American Psychiatric Association, 2013; Willcutt, 2012). These symptoms typically manifest as difficulties in staying alert, resisting distractions, memorizing information, organizing, planning, and monitoring actions and tasks, estimating time, and persisting engagement in tasks and activities (Nigg & Barkley, 2014; Willcutt, 2015). Evidence suggests that roughly one fifth of children in community-based samples have moderate to high and persistent levels of inattention symptoms until late adolescence (Döpfner, 2015; Vergunst et al., 2019). This is of concern since inattention symptoms have been linked to suboptimal and problematic functioning in secondary school, including low perceived academic competence (Scholtens et al., 2013), avoidance orientation in learning situations (Duchesne et al., 2014), rejection and victimization by classmates (Ji et al., 2019), and academic impairment in math and reading (Breslau et al., 2009).

Surprisingly, students’ PNS at school has been largely overlooked in the ADHD empirical literature. To our knowledge, only two studies have so far focused on this topic. In one of these studies (Rogers & Tannock, 2018), students in grades 1 to 4 that have high levels of ADHD symptoms reported lower perceptions of autonomy support, competence, and relatedness to their teacher, compared to their asymptomatic peers. In the second study (Oram et al., 2020), self-reported ADHD symptomatology in a sample of undergraduate students was positively and moderately associated with autonomy, competence, and relatedness need frustration.

Despite preliminary evidence suggesting a possible link between inattention symptoms and low need satisfaction in both younger and older students, further investigation is needed, especially with secondary school students. To this end, the primary-secondary school transition appears a suitable time to examine the prospective contribution of inattention symptoms on PNS. Students migrate to a school environment that is frequently perceived as more controlling, performance-oriented, and impersonal, which may hamper their developmental needs (Eccles et al., 1993; Eccles & Roeser, 2011). In this context, it could be particularly challenging for inattentive students who are prone to executive deficits, low academic performance, and social exclusion to fully meet their basic psychological needs at school.

## Teachers’ Involvement as a Potential Protective Factor

SDT argues that students with vulnerabilities have the same basic psychological needs as their peers without vulnerabilities and that interpersonal behaviors elicited by teachers in the classroom are important to support these needs (Ryan & Deci, 2017). Some of these behaviors are grouped under a dimension called *involvement* (Skinner & Belmont, 1993; Stroet et al., 2013, 2015). Teachers’ involvement refers to behaviors characterized by the allocation of emotional and material resources such as spending time with students, listening, showing genuine interest, and providing emotional support and encouragement. To date, SDT research on teachers’ interpersonal behaviors mainly focused on autonomy support, structure, and control (Ryan & Deci, 2017). However, there is evidence that their involvement also benefits students’ PNS in secondary school setting. In essence, studies indicated that teachers’ involvement (Tucker et al., 2002; Zhang et al., 2011), social support from teachers (Tian et al., 2016), positive teacher-student relationship (Bakadorova & Raufelder, 2018), and feeling a sense of belonging with teachers (Froiland et al., 2019) were positively related to students’ PNS.

Based on the above research findings, it is reasonable to assume that teachers’ involvement could be pivotal for students struggling with attention problems. Indirect support for this proposition is provided by research conducted with at-risk students. For example, at-risk kindergarten students (e.g., exhibiting problem behaviors) exposed to a highly emotionally supportive teacher in first grade had comparable achievement performance as students considered at low risk (Hamre & Pianta, 2005). In contrast, at-risk students whose teachers manifested low to moderate levels of support achieved at lower levels than those with low-risk status. Another investigation showed that elementary students with high levels of externalizing or internalizing problems performed better in reading when experiencing a positive teacher-student relationship (Baker, 2006). Conversely, students who displayed similar problems and had a negative relationship with their teacher performed at a lower level. Finally, a study using a sample of at-risk high school students (e.g., showing severe behavioral problems) who participated in a teacher-student mentoring program (Larose et al., 2020) found that a positive bond with one’s teacher positively predicted both motivation and persistence intentions for students with low-mastery orientation (low focus on understanding and improvement). For those with a high-mastery orientation, predictive associations were of weaker magnitude. Considering these results, it appears relevant to investigate the moderating role of teachers’ involvement in the association between students’ inattention symptoms and PNS at school.

## Possible Confounders

Three potential confounding variables were taken into account in this study, namely gender, anxiety, and aggression. Studies indicated that in adolescence, girls have fewer inattention symptoms (Döpfner, 2015; Vergunst et al., 2019) and report higher PNS across the high school years (Ratelle & Duchesne, 2014) than boys. In addition, adolescents’ self-reported attention problems tend to co-occur with anxiety and antisocial behaviors such as aggression (Connors et al., 2012; Schaughency et al., 1994). Some studies have also shown that anxiety (Yu et al., 2016) and aggression in adolescence (Kuzucu & Şimşek, 2013) were negatively associated with PNS. By controlling for these variables, the unique contribution of attention problems in the prediction of PNS would be better estimated.

## The Present Study

Guided by SDT, this prospective study pursued two main goals. The first goal was to evaluate the predicting role of students’ inattention symptoms at the end of elementary school on changes in PNS during the secondary school transition. Based on recent research, it was hypothesized that inattention symptoms would predict a decline in PNS at school, above the contribution of gender, anxiety symptoms, and aggressive behaviors. The second goal was to test the moderating role of teachers’ involvement in the first year of secondary school (Secondary 1) on the predictive relationship between inattention symptoms and changes in PNS. Consistent with SDT and studies with at-risk students, inattention symptoms were expected to more strongly predict decreases in PNS when students perceived their teacher’s involvement in their classroom as low. Teachers’ involvement was assessed in mathematics and language classes. In the Quebec school system, these subjects occupy the most teaching time in the curriculum (Ministère du Travail, de l’Emploi et de la Solidarité sociale, 2020). No specific hypotheses were formulated for these subjects.

## Method

### Participants and Procedure

Data were collected from a longitudinal study in the French-speaking province of Quebec aimed at describing and understanding student transition, adjustment, and persistance in school. A total of 688 students in their final year of elementary school (Grade 6) took part in the study. The sample (46% male) was recruited with the collaboration of the Quebec Ministry of Education, which provided a random list of students stratified by gender, socioeconomic status, and geographic area (urban or rural). At the first measurement time point, their mean age was 11.82 years (*SD*_age_ = 0.48). Approximately 93% of them were born in Quebec, 98% had French as their mother tongue, and 71% lived with both parents. They were mostly from middle-class backgrounds, as indicated by the median family income reported by mothers ($50,000–$59,000 CAD). For the same period, the median household income in Quebec was $57,000 CAD (Statistics Canada, 2021). Each year (April–May), all participants completed a questionnaire battery at home (either online or in paper version). The current study focused on the first two waves of the study (Grade 6 and Secondary 1).

### Measures

#### Inattention symptoms

In Grade 6, students’ inattention symptoms were assessed by their mothers using the Attention problems subscale of the Child Behavior Checklist (CBCL; Achenbach & Rescorla, 2001). Four items (i.e., can’t concentrate and can’t pay attention for long, daydreams or gets lost in their thoughts, impulsive or acts without thinking, and poor schoolwork) were selected on the basis of their severity levels and loading factors (e.g., Hudziak et al., 1999; Lengua et al., 2001). Each item was measured using a 3-point scale ranging from 1 (never applies) to 3 (frequently applies).

#### Psychological need satisfaction at school (PNS)

In Grade 6 and Secondary 1, three subcales were used to measure students’ perceived PNS at school: (1) the Academic subscale of the Perceived Self-Determination in Life Domains Scale (Blais & Vallerand, 1991) assessed *autonomy satisfaction* (three items; e.g., “I go to school out of personal choice”); (2) the Academic subscale of the Perceived Competence in Life Domains Scale (Losier et al., 1993) measured *competence satisfaction* (three items; e.g., “At school, I have developed very good competences as a student”); (3) and the Intimacy subscale of the Need for Relatedness Scale (Richer & Vallerand, 1998) assessed *relatedness satisfaction* (three items; e.g., “In my relationships with my classmates, I feel close to them”). Students were asked to indicate their level of agreement on a Likert-type scale ranging from 1 (do not agree at all) to 7 (strongly agree). The psychometric qualities of these scales were supported in previous studies (e.g., Losier et al., 1993; Richer & Vallerand, 1998).

#### Teacher’s involvement

In Secondary 1, students’ perceptions of the teacher’s involvement in mathematics and French were assessed with the Personalization subscale of the Individualized Classroom Environment Questionnaire (Fraser & Fisher, 1986). This 5-item subscale measures the extent to which the teacher is perceived as available and accessible in the classroom (e.g., “the mathematics/French teacher takes a personal interest in each student”). Students were asked to indicate their level of agreement on a 5-point scale ranging from 1 (do not agree at all) to 5 (strongly agree). This subscale was found to be reliable and valid (e.g., Fraser, 1991).

#### Anxiety symptoms

In Grade 6, students self-reported anxiety symptoms were assessed with the French-Canadian version (Turgeon & Chartrand, 2003) of the worry/oversensitivity subscale of the Revised Children’s Manifest Anxiety Scale (Reynolds & Richmond, 1997). It contains 12 self-rated items (e.g., “I worry a lot of the time”) using a yes/no format. In this study, six items were retained based on their factor loading (see Turgeon & Chartrand, 2003).

#### Aggression

Mothers assessed their child’s manifestations of aggression in Grade 6 using the Aggressive behavior subscale of the CBCL. They were asked to answer three items (i.e., threatens people; cruelty, bullying, or meanness to others; and gets in many fights) using a 3-point scale ranging from 1 (never applies) to 3 (frequently applies). The reliability of this subscale was supported in previous studies (e.g., Achenbach & Rescorla, 2001).

### Statistical Analyses

#### Model estimation

We tested our hypotheses using a structural equation modeling approach using Mplus (version 8.4; Muthén & Muthén, 1998–2020). The factorial structure of the latent constructs was examined by exploratory structural equation modeling (ESEM; Marsh et al., 2009). This method allows observed indicators to freely load on multiple latent factors (e.g., teachers’ involvement in mathematics and French). Given the number of items (41) and factors (11) estimated, two measurement models were analyzed. The first included control variables (anxiety, aggression), the predictor (inattention), and predicted variables (PNS in Grade 6 and Secondary 1). In the second model, the factorial structure of the moderators (teachers’ involvement in mathematics and French) was examined. The adequacy of the models was assessed with the Bentler comparative fit index (CFI), the Tucker-Lewis index (TLI), and the root mean squared error of approximation (RMSEA) with 90% confidence intervals. For CFI and TLI, values greater than or equal to 0.90 indicate an acceptable fit to the data, and a RMSEA value below 0.08 supports a well-fitting model (e.g., Hu & Bentler, 1999).

An ESEM-within-CFA (confirmatory factor analysis) was applied for the regression model (Marsh et al., 2009). This method allows a first order ESEM solution to be reexpressed via CFA solution. The maximum likelihood robust (MLR) estimator was used, which produce corrected standard errors when data is not normally distributed (Enders & Bandalos, 2001). For each psychological need, latent difference scores (LDS; McArdle, 2009) were created. In this procedure, latent score at a given wave (e.g., autonomy satisfaction in Secondary 1) is a function of the latent score at the preceding wave (e.g., autonomy satisfaction in Grade 6) plus change between waves (e.g., autonomy satisfaction in Secondary 1 to autonomy satisfaction in Grade 6). Hence, this decomposition allows capturing interindividual differences in intraindividual change at the latent level, corrected for measurement errors (Geiser et al., 2010). All other latent constructs included in the model (i.e., inattention, anxiety, aggression, and teachers’ involvement) were measured by their corresponding observed indicators. Finally, six latent interactions were estimated. In addition to inattention × teachers’ involvement (in math and French), anxiety × teachers’ involvement (in math and French), and aggression × teachers’ involvement (in math and French) interactions were also added as covariates.

#### Missing data

The proportion of incomplete data across the school transition ranged from 29% to 34%. Missingness was handled with the full-information maximum likelihood (FIML) estimator (Enders, 2001; Muthén & Muthén, 1998–2020), which is recommended over deletion techniques (listwise and pairwise) and mean substitution (e.g., Baraldi & Enders, 2010; Myers, 2011).

## Results

### Preliminary Analyses

#### Measurement models

Fit indices of the first measurement model (anxiety, aggression, inattention, PNS in Grade 6 and Secondary 1) suggested good model fit, χ^2^ (222) = 375.25, *p* < .01; CFI = 0.96; TLI = 0.92; RMSEA = 0.03, 90% CI [0.03, 0.04]). Except for one item (λ = .26), all standardized factor loadings varied between 0.31 and 0.88 and loaded more strongly on their assigned construct. For the second model (teachers’ involvement in mathematics and French), the yielded solution was excellent (χ^2^ (21) = 47.14, *p* < .01; CFI = 0.98; TLI = 0.96; RMSEA = 0.05, 90% CI [0.03, 0.07]). All factor loadings ranged between 0.33 and 0.80 and loaded more strongly on their target factors.

#### Correlations

Table 1 presents correlations among latent factors. Most of the relationships are in the expected direction with small-to-large effect sizes. Three sets of results are noteworthy. First, inattention symptoms were negatively related to PNS in Grade 6 and Secondary 1. Second, teachers’ involvement in both mathematics and French classes were positively associated with PNS in Secondary 1. Third, autonomy satisfaction was found to be more stable during the secondary school transition than competence and relatedness satisfaction.

**Table 1.** Correlations Among Latent Factors (*N* = 688), Descriptive Statistics, and Reliability Coefficients (ω).

	1	2	3	4	5	6	7	8	9	10	11
1. Autonomy (*T*1)	—										
2. Competence (*T*1)	.42***	—									
3. Relatedness (*T*1)	.08	.37***	—								
4. Inattention (*T*1)	−.29***	−.35***	−.08	—							
5. Aggressivity (*T*1)	−.08	.01	−.07	.31***	—						
6. Anxiety (*T*1)	−.17**	−.19***	−.13*	.09	.01	—					
7. Autonomy (*T*2)	.72***	.23**	.13*	−.40***	−.16	−.08	—				
8. Competence (*T*2)	.25***	.51***	.06	−.38***	−.09	−.09	.43***	—			
9. Relatedness (*T*2)	.11	.10	.44***	−.12	−.10	−.10	.22***	.20***	—		
10. Teacher’s involvement— French (*T*2)	.17*	.15*	.06	−.02	.00	−.02	.20***	.26***	.21***	—	
11. Teacher’s involvement—Math (*T*2)	.11	.12	−.02	−.01	.01	−.08	.09	.21***	.16**	.53***	—
*M*	5.09	5.66	4.98	1.41	1.21	1.40	5.08	5.46	4.92	3.54	3.47
*SD*	0.06	0.04	0.06	0.02	0.01	0.01	0.07	0.05	0.07	0.04	0.04
Scale range	1−7	1−7	1−7	1−3	1−3	1−2	1−7	1−7	1−7	1−5	1−5
ω	.63	.70	.83	.79	.70	.66	.71	.77	.86	.76	.74

*Note. T*1 = (Time 1/Grade 6); *T*2 = (Time 2/Secondary 1).

* *p* < .05. **p < .01. ***p < .001.

#### Gender differences

Finally, gender differences were examined by regressing latent factors (11) on gender. Small-to-medium statistically significant associations (*p* < .01) were found for six out of eleven variables. Specifically, the results showed that being an adolescent girl was positively related to higher autonomy satisfaction (β_Grade 6_ = .30, 95% CI [0.20, 0.39]; β_Secondary 1_ = .30, 95% CI [0.20, 0.39]), competence satisfaction (β_Grade 6_ = .13, 95% CI [0.04, 0.22]), relatedness satisfaction (β_Secondary 1_ = .31, 95% CI [0.22, 0.40]), and anxiety (β_Grade 6_ = .22, 95% CI [0.12, 0.31]), and linked to fewer inattentive symptoms (β_Grade 6_ = −.16, 95% CI [−0.28, −0.04]). These findings supported the relevance of controlling for the contribution of gender in main analyses.

### Main Analyses

Table 2 summarizes the results of the regression model. The hypothesis concerning the direct contribution of inattention symptoms and teachers’ involvement was confirmed for two of the three psychological needs. More specifically, inattention in Grade 6 predicted a moderately weak decline in autonomy and competence satisfaction between Grade 6 and Secondary 1, after adjusting for gender, PNS, anxiety, and aggression in Grade 6. Conversely, teachers’ involvement in both mathematics and French classes predicted a small decrease in competence and relatedness satisfaction during the secondary school transition, beyond control variables. The explained variance (R^2^) for each LDS is moderate (LDS_autonomy_ = 0.22; LDS_competence_ = 0.28; LDS_relatedness_ = 0.35).

**Table 2.** Regression Predicting Latent Changes in Psychological Need Satisfaction (*N* = 688).

Factors	LDS autonomy		LDS competence		DS relatedness	
	β	*SE*	β	*SE*	β	*SE*
Gender^a^	.13*	0.06	.04*	0.05	−.30***	0.04
Autonomy (*T*1)	−.34***	0.06	−.05***	0.07	−.04***	0.06
Competence (*T*1)	−.16*	0.07	−.46***	0.07	−.12	0.07
Relatedness (*T*1)	.10	0.06	−.12*	0.06	−.47***	0.04
Inattention (*T*1)	−.21***	0.07	−.20**	0.08	−.03	0.05
Aggression (*T*1)	−.01	0.07	−.01	0.08	−.04	0.06
Anxiety (*T*1)	−.01	0.05	−.01	0.06	−.11*	0.05
Teacher’s involvement—French (*T*2)	.10	0.06	.17**	0.06	.15***	0.04
Teacher’s involvement—Math (*T*2)	.02	0.06	.12*	0.06	.13**	0.05
Inattention (*T*1) × involvement—French (*T*2)	.18*	0.07	.10	0.09	−.01	0.06
Inattention (*T*1) × involvement—Math (*T*2)	−.06	0.06	.04	0.09	.06	0.06
Aggression (*T*1) × involvement—French (*T*2)	−.17	0.09	.04	0.10	.06	0.06
Aggression (*T*1) × involvement—Math (*T*2)	.16	0.09	−.02	0.09	−.06	0.09
Anxiety (*T*1) × involvement—French (*T*2)	−.10	0.07	−.07	0.07	−.08	0.06
Anxiety (*T*1) × involvement—Math (*T*2)	−.07	0.07	−.05	0.07	.02	0.07

*Note. T*1 = (Time 1/Grade 6); *T*2 = (Time 2/Secondary 1). LDS = latent difference score.

a Girls serve as the reference group.

* *p* < .05. **p < .01. ***p < .001.

**Figure 1.**
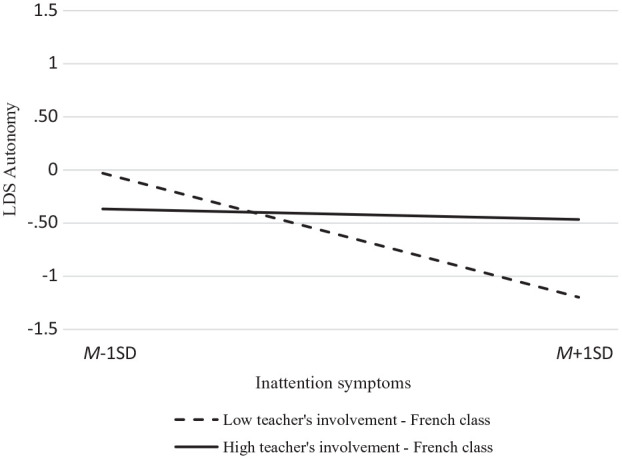
Inattention (Grade 6) × teacher’s involvement (Secondary 1) interaction effect predicting change in autonomy satisfaction.

The moderating role of teachers’ involvement was supported only for changes in autonomy satisfaction where a statistically significant interaction was found between inattention and teachers’ involvement in French (see Table 2). As illustrated in Figure 1, regression slopes indicated that students who have more inattention symptoms in Grade 6 (i.e., 1 *SD* below the mean) experience a lower decline in autonomy satisfaction across the school transition if they perceived their language teacher as highly involved in Secondary 1 (β = −.03, *p* < .01). Similarly at-risk students who perceived their teacher as uninvolved in Secondary 1 experienced a steeper decrease in autonomy satisfaction during this transition (β = −.39, *p* < .01).

Regarding covariates, the results showed that autonomy satisfaction in Grade 6 was linked with greater decline in autonomy need satisfaction during high school transition. Moreover, competence satisfaction in Grade 6 was associated with greater decrease in competence need satisfaction and, to a lesser extent, with decline in autonomy need satisfaction. Similar associations were observed for relatedness satisfaction in Grade 6 in the prediction of relatedness and competence need satisfaction change, respectively. Anxiety symptoms in Grade 6 also predicted a small decline in relatedness satisfaction. Finally, being an adolescent girl was associated with a small increase in autonomy satisfaction and a moderate decrease in relatedness satisfaction.

## Discussion

The current study is the first to examine the prospective relationship between inattention symptoms in Grade 6 and PNS change during the transition to secondary school as well as to test the moderating role of teachers’ involvement in this association. Results revealed that inattention predicted a moderately weak decline in autonomy and competence satisfaction but that it was unrelated to changes in relatedness satisfaction. A moderating effect was also detected where inattention predicted a smaller decline in autonomy satisfaction when students perceived their teachers as highly involved, compared to their peers with an uninvolved teacher. All relationships were adjusted for multiple potential confounders (need satisfaction at baseline, anxiety, aggression, and gender). The scientific and applied implications of these findings are discussed below.

### Inattention Symptoms and Changes in PNS

The observed pattern between inattention and need satisfaction (autonomy and competence) is consistent with previous studies suggesting that basic psychological needs were more likely to be unfulfilled among elementary (Rogers & Tannock, 2018) and undergraduate (Oram et al., 2020) students with attention problems. The present study adds to this empirical literature by using a sample of students transitioning to high school, a prospective design with two measurement times over a 1-year interval, and a LDS approach to assess change in PNS.

The fact that students’ inattention symptoms predicted a decline in autonomy satisfaction during the secondary school transition can be explained by their difficulty in mobilizing cognitive functions that are crucial for the learning process. In this regard, it is well documented that memorization, inhibitory control of automatic responses, planning, processing speed, and flexible sequencing of actions are often impaired in youth with attention problems (e.g., Willcutt, 2015). While SDT claims that the presence of individual vulnerabilities may undermine PNS (Ryan & Deci, 2017), it is reasonable to believe that students with difficulties such as selecting and using effective strategies, monitoring comprehension and goal-directed behaviors, resisting distractions, and persevering could feel that their thoughts and actions in learning situations are not fully volitional and self-endorsed. At the beginning of secondary school, this feeling could intensify as this new context can provide fewer opportunities for students’ autonomy (e.g., make decisions and choices) than in primary school (Eccles et al., 1993).

Although the above executive deficits hypothesis could also apply to the relationship between inattention and decreased competence satisfaction, other factors could also be involved. In this regard, academic achievement appears to be a prime candidate since inattention symptoms can predict lower student performance (Breslau et al., 2009). Actual performance is seen as a reliable indicator by which students assess their own ability to produce desired outcomes in achievement situations (e.g., Usher & Schunk, 2018). For students with inattention symptoms at the end of elementary school, poor performance in the first formal assessments in secondary school could progressively alter their competence satisfaction by confirming that academic challenges in this unfamiliar setting are difficult to meet and require cognitive resources they have not fully developed. This assumption appears plausible based on findings that ADHD symptoms in secondary school were negatively related with achievement and perceived academic competence (Scholtens et al., 2013). The decreasing competence satisfaction of students with attention problems could even be fueled in highly performance-oriented classrooms that encourage peer comparison (e.g., Eccles et al., 1993; Usher & Schunk, 2018). Poor performance in an environment that values individual success and achievement could lead to a negative self-image and thwart students’ need for competence in school.

Contrary to what was hypothesized, inattention symptoms were not prospectively associated with change in relatedness satisfaction. Partial support had been obtained for this hypothesis (Oram et al., 2020; Rogers & Tannock, 2018), but these studies differed from the current investigation in several methodological aspects (e.g., age of participants, research design, analytical strategies, ADHD symptoms considered, and measurement of psychological needs). Beyond these disparities, the absence of a link for attention problems could be explained by their concomitance with anxiety symptoms. This study is the first to show that anxiety predicted a decline in relatedness satisfaction during the secondary school transition, while controlling for confounders (including inattention). Here, anxiety focused primarily on students’ concerns (e.g., worrying about what others think). When excessive, such concerns could lead to the adoption of self-protective behaviors (e.g., avoiding peers or activities in school) that would limit social exploration and bonding. Considering that many adolescent students exhibit both inattention and anxiety symptoms (e.g., Connors et al., 2012), it could be argued that part of the decline in relatedness satisfaction at school would be driven by their inclination to worry about social situations rather than by their inattention or distractibility in academic tasks or activities. Further research is needed to confirm this proposition.

### Teachers’ Involvement and Changes in PNS

The results of this study support SDT’s postulate that teachers’ involvement contribute to promoting PNS at school for all students. Most importantly, for those with attention problems, they highlighted the benefits of this involvement in autonomy satisfaction. While a handful of past studies reported similar results (e.g., Bakadorova & Raufelder, 2018; Froiland et al., 2019), none had yet (1) explored the protective role of involvement on inattention, (2) evaluated PNS change during high school transition, (3) measured this change using a robust analytical method (LDS), or (4) considered multiple covariates.

According to SDT, an involved teacher is one who devotes time, interest, and support to their students (e.g., Stroet et al., 2013). Teachers’ involvement can help them gain their students’ confidence and encourage the expression of their emotions and educational needs. In such a relational context, students could feel understood, valued, supported, and cared for, which would in turn contribute to fulfilling their needs for competence (experiencing a sense of self-efficacy) and relatedness (experiencing connection). For students with attention problems, teachers’ involvement appears to be important for their autonomy satisfaction. An involved teacher might be well positioned to detect their cognitive vulnerabilities (e.g., distractibility and disorganization) and to address them by providing scaffolding, constructive feedback, and encouragement (e.g., Ryan & Deci, 2017). As a result, students could feel less controlled or pressured because the teacher attempts to compensate for their deficits (Ryan & Deci, 2017). Ultimately, this experience of trust and support with the teacher could transcend the classroom context and contribute more broadly to the satisfaction of their need for autonomy at school.

### Implications for Practice

Researchers (e.g., DuPaul & Eckert, 1997) have made recommendations regarding educational principles that may compensate for attention deficits and improve academic performance of children and adolescents with ADHD (e.g., shorter work periods, contingency management, and peer tutoring). However, other modalities could be necessary or complementary to support the PNS of students with inattention symptoms, especially during their transition to secondary school. In this study, three key findings suggest targeting teachers’ involvement. First, this category of interpersonal behaviors attenuated the negative relationship between inattention and autonomy satisfaction (protective effect). Second, it also contributed directly and positively to competence satisfaction, over and above the negative contribution of inattention symptoms (compensatory effect). Third, teachers’ involvement was positively associated to relatedness satisfaction, beyond the negative contribution of anxiety, a co-occurring condition frequently identified with inattention (e.g., Connors et al., 2012). Taken as a whole, these findings point to the importance of providing teachers with training programs designed to develop attitudes (e.g., listening and empathy) and know-how (e.g., support and encouragement) that will communicate their involvement to their students, especially those with inattention symptoms. To this end, a multiple case-study design rooted in SDT suggests that it is possible to improve the involvement of teachers working with vulnerable students (students with congenital and acquired deaf blindness) following individual homework assignments and video coaching (Haakma et al., 2017).

### Limitations and Recommendations for Future Research

Despite its many strengths (i.e., robust theoretical framework, representative sample, multiple informants, prospective design, sophisticated statistical modeling, and control for important variables), this study has limitations that need to be considered when interpreting findings, and that should be addressed in future research. First, the use of a prospective-correlational design does not allow making causal inferences on the directionality of relations between variables. Here, inattention symptoms were proposed as determinants of PNS at school. However, attentional processes have also been predicted by PNS experienced in lessons (Oram et al., 2020). Further research should explore the cross-lagged temporal relationship between these constructs by adding a third wave (e.g., de Hann et al., 2019). Second, the present study used a community sample of secondary school students that do not allow generalization to clinical populations. Further research is needed to confirm the obtained pattern of findings with students that received a clinical diagnosis of ADD or ADHD. Third, inattention symptoms were assessed from mothers’ perspective and were not contextualized to school, as was PNS. Future research should consider the level of specificity with which inattention and PNS are measured (school-school vs. home-school). Fourth, related to the above, combining parent report of attentional symptoms with a neuropsychological test (e.g., Tests of Variables of Attention) would have counterbalanced for possible bias in symptoms reports. Five, information on participants’ formal psychological diagnoses and psychiatric medication use was not collected. Future research could exploit these as control variables to better isolate the contribution of inattention symptoms. Finally, teachers’ involvement was considered in this study as potential moderator. According to the SDT literature, two other categories of teacher behaviors contribute to PNS, namely autonomy support and structure (Ryan & Deci, 2017). Future investigation could consider these crucial behaviors in their methodological design and analysis.

## Conclusion

The passage from primary to secondary school is characterized by significant academic and social changes. In this study, it was hypothesized that students with attention problems would find it more challenging to fulfill their psychological needs during this period. The results showed that inattention symptoms predicted a decrease in need satisfaction for autonomy and competence, but not for relatedness. These prospective associations were adjusted for multiple confounders (gender, anxiety, aggression, and need satisfaction at baseline). Results also suggested that teachers’ involvement (1) buffers the contribution or inattention on autonomy satisfaction and (2) directly predicted increases in competence satisfaction. These findings highlight the importance of teachers’ involvement for promoting their students PNS when they are coping with inattention symptoms throughout a pivotal school transition.
